# Population-Based Estimates of Health Care Utilization and Expenditures by Adults During the Last 2 Years of Life in Canada’s Single-Payer Health System

**DOI:** 10.1001/jamanetworkopen.2020.1917

**Published:** 2020-04-01

**Authors:** Laura C. Rosella, Kathy Kornas, Catherine Bornbaum, Anjie Huang, Tristan Watson, Jennifer Shuldiner, Walter P. Wodchis

**Affiliations:** 1 Dalla Lana School of Public Health, University of Toronto, Toronto, Ontario, Canada; 2 ICES, Toronto, Ontario, Canada; 3 Public Health Ontario, Toronto, Ontario, Canada; 4 Health and Rehabilitation Sciences, Faculty of Health Sciences, Western University, London, Ontario, Canada; 5 Institute of Health Policy, Management, and Evaluation, University of Toronto, Toronto, Ontario, Canada; 6 Institute for Better Health, Trillium Health Partners, Mississauga, Ontario, Canada

## Abstract

**Importance:**

Measuring health care utilization and costs before death has the potential to initiate health care improvement.

**Objective:**

To examine population-level trends in health care utilization and expenditures in the 2 years before death in Canada’s single-payer health system.

**Design, Setting, and Participants:**

This population-based cohort included 966 436 deaths among adult residents of Ontario, Canada, from January 2005 to December 2015, linked to health administrative and census data. Data for deaths from 2005 to 2013 were analyzed from November 1, 2016, through January 31, 2017. Analyses were updated from May 1, 2019, to June 15, 2019, to include deaths from 2014 and 2015.

**Exposures:**

Sociodemographic exposures included age, sex, and neighborhood income quintiles, which were obtained by linking decedents’ postal codes to census data. Aggregated Diagnosis Groups were used as a general health service morbidity-resource measure.

**Main Outcomes and Measures:**

Health care services accessed for the last 2 years of life, including acute hospitalization episodes of care, intensive care unit visits, and emergency department visits. Total health care costs were calculated using a person-centered costing approach. The association of area-level income with high resource use 1 year before death was analyzed with Poisson regression analysis, controlling for age, sex, and Aggregated Diagnosis Groups.

**Results:**

Among 966 436 decedents (483 038 [50.0%] men; mean [SD] age, 76.4 [14.96] years; 231 634 [24.0%] living in the lowest neighborhood income quintile), health care expenditures increased in the last 2 years of life during the study period (CAD$5.12 billion [US $3.83 billion] in 2005 vs CAD$7.84 billion [US $5.86 billion] in 2015). In the year before death, 758 770 decedents (78.5%) had at least 1 hospitalization episode of care, 266 987 (27.6%) had at least 1 intensive care unit admission, and 856 026 (88.6%) had at least 1 emergency department visit. Overall, deaths in hospital decreased from 37 984 (45.6%) in 2005 to 39 474 (41.5%) in 2015. Utilization in the last 2 years, 1 year, 180 days, and 30 days of life varied by resource utilization gradients. For example, the proportion of individuals visiting the emergency department was slightly higher among the top 5% of health care users compared with other utilization groups in the last 2 years of life (top 5%, 45 535 [94.2%]; top 6%-50%, 401 022 [92.2%]; bottom 50%, 409 469 [84.7%]) and 1 year of life (top 5%, 43 007 [89.0%]; top 6%-50%, 381 732 [87.8%]; bottom 50%, 380 859 [78.8%]); however, in the last 30 days of life, more than half of individuals in the top 6% to top 50% (223 262 [51.3%]) and bottom 50% (288 480 [59.7%]) visited an emergency department, compared with approximately one-third of individuals in the top 5% (16 916 [35.0%]). No meaningful associations were observed in high resource use between individuals in the highest income quintile compared with the lowest income quintile (rate ratio, 1.02; 95% CI, 0.99-1.05) after adjusting for relevant covariates.

**Conclusions and Relevance:**

In this study, health care use and spending in the last 2 years of life in Ontario were high. These findings highlight a trend in hospital-centered care before death in a single-payer health system.

## Key Points

**Question:**

What are the population-level trends in health care utilization and expenditures in the 2 years before death among adults in Ontario, Canada?

**Findings:**

This cohort study found that health care expenditures in the last 2 years of life increased in Ontario from CAD$5.12 billion in 2005 to CAD$7.84 billion in 2015, and the intensity of health care utilization and deaths in hospital varied by resource utilization gradients.

**Meaning:**

In this study, the observed trends demonstrated that costs and hospital-centered care before death are high in Ontario.

## Teaser

This cohort study examines population-level trends in health care utilization and expenditures in the 2 years before death among adults in Canada’s single-payer health system.

## Introduction

Similar to those in other high-income countries, health care utilization and costs in Canada are expected to increase because of an expanding and aging population.^1^ A large proportion of these costs are incurred toward the end of life, with multiple studies demonstrating that health care utilization in the final months of life accounts for a substantial share of health care expenditures in comparison with other points in an individual’s life.^2,3,4,5^ In addition, most spending is concentrated in small groups of the population, who are characterized as high-cost users.^6,7^ Studies have shown that high-intensity medical care at the end of life can produce poor outcomes,^8,9,10^ can be associated with poor quality of life,^11^ and may conflict with patient preferences.^9,12^ To meet the growing needs of an aging population, a deeper understanding of the determinants and patterns of health care utilization and costs prior to death is required.

Most studies examining health care utilization prior to death have focused on a single aspect of care (eg, palliative services)^13^ or were specific to a particular cause of death.^14,15,16,17,18^ To our knowledge, few studies have examined health care use and costs at a population level and across an array of health sectors.^5,19^ Despite its potential to inform health care service delivery and improvement, evidence on health care utilization and cost patterns before death in a Canadian context is limited. A recent population-based study that examined health care expenditures in Ontario, Canada, from 2010 to 2013^19^ reported that decedents who constituted less than 1% of the population consumed 10% of Ontario’s total health care budget, demonstrating that health care utilization occurs disproportionately. Using comprehensive multilinked mortality files, we analyzed population-level trends in health care utilization and expenditures prior to death in Ontario’s single-payer health system by looking at overall trends for more than a decade and by gradients of cost (ie, patients in the top 5%, top 6%-50%, and bottom 50% of health care costs).

## Methods

### Study Design

This retrospective cohort study used multiple linked vital statistics, population files, and health administrative data held at ICES to examine all deaths occurring in Ontario between January 2005 and December 2015. These data sets were linked using unique encoded identifiers and analyzed at ICES, an independent, nonprofit research institute whose legal status under Ontario’s health information privacy law allows it to collect and analyze health care and demographic data, without consent, for health system evaluation and improvement. This study received ethical approval from the University of Toronto’s Health Sciences research ethics board and the institutional review board at Sunnybrook Health Sciences Centre, Toronto, Canada. This study followed the Strengthening the Reporting of Observational Studies in Epidemiology (STROBE) reporting guideline.

### Study Population

Data for all deaths registered in the province of Ontario were obtained from the Office of the Registrar General-Deaths (ORG-D) file. The ORG-D is linked to the Registered Persons Database (RPDB), which contains basic demographic information for those who have ever received an Ontario health card number for the province’s universal health care system (overall linkage rate, 96.5%).^20^ The study cohort consisted of all deaths registered in the ORG-D between January 1, 2005, and December 31, 2015, that were linked to the RPDB record (N = 966 436). Those who had an invalid Ontario health card number on their death date (n = 4433), were not residents of Ontario (n = 252), or were younger than 18 years (n = 8768) were excluded.

### Measures

We examined health care utilization prior to death according to several sociodemographic exposures. Sex and age data were obtained from the RPDB. Categories for age at time of death were 18 to 24 years, 25 to 34 years, 35 to 44 years, 45 to 54 years, 55 to 64 years, 65 to 74 years, 75 to 85 years, and older than 85 years. Ecological-level measures of income and education status were estimated using data from the 2006 Canadian census^21^ and were applied to individuals according to the dissemination area, which represents the smallest geographic census area in which the individual resided. Based on their postal code at the time of death, individuals were assigned to a dissemination area. Education was characterized as the proportion of individuals who completed high school in a given dissemination area. Individuals were grouped into income and education quintiles ranging from 1 (lowest 20% of income or education) to 5 (highest 20% of income or education).

As a general health service morbidity-resource measure, we used The Johns Hopkins Adjusted Clinical Group system version 10.0.1 Aggregated Diagnosis Group (ADG) scores, a person-focused, diagnosis-based method of categorizing individuals’ illnesses.^22^ Aggregated Diagnosis Groups have been validated for health services research use in Ontario^23^ and were calculated for the 2 years prior to death.

We measured health care utilization and services accessed for 2 years, 1 year, 180 days, and 30 days before death. Hospitalization episodes of care and intensive care unit (ICU) visits were obtained from the Discharge Abstract Database. An acute hospitalization episode was defined as either an admission to an acute care setting from which the patient was discharged or a continuous sequence of hospital stays in different hospitals to which the patient was transferred. Transfers between 2 different institutions were defined using both the timing between admissions and transfer flags on either record. Specifically, the following situations were defined as a transfer: (1) any admission within 6 hours of the previous discharge, (2) any admission within 12 hours of the previous discharge in which the type of institution transferred from or to was type 1 (ie, acute care), or (3) any admission within 48 hours of the previous discharge in which the number of the institution transferred from matched the number of the institution or the institution transferred to. Length of stay for episodes of care and ICU visits were calculated by subtracting the date of the latest discharge from the date of the earliest admission. Emergency department visits were obtained from the National Ambulatory Care Reporting System and were counted as 1 claim per patient per registered day. Physician services (primary care and specialist) were obtained from the Ontario Health Insurance Plan claims database. A physician visit was counted as 1 claim per patient per service day per physician. Physician specialties listed as family practice and general practice, community medicine, and pediatrics were considered primary care visits. All other physician specialties were considered a specialist visit. Death in hospital was identified in the Discharge Abstract Database if a hospital discharge disposition code was recorded as died, indicating a death in hospital. In 423 580 of 427 859 in-hospital deaths (99.0%), the death date in ORG-D and hospital discharge date with a code indicating died in the Discharge Abstract Database were within 1 day apart.

We calculated comprehensive per-person health care costs for the time proceeding death (last 2 years of life). Annual health care utilization and costs were calculated from the health care payee perspective, using administrative data from across health care sectors, including inpatient hospital stay, emergency department visits, same day surgery, stays in complex continuing care hospitals and inpatient rehabilitation, inpatient psychiatric admissions, physician payments for patient visits and community laboratory tests, and prescriptions filled for individuals eligible for the Ontario Drug Benefit Plan. A person-centered costing macro was used to calculate total annual health care spending; the costing methodology has been described elsewhere.^24^ Expenditures were calculated in Canadian dollars for the year 2015. Individuals were categorized in resource utilization groups (top 5%, top 6%-50%, and bottom 50%) based on the total health care costs in the last year of their lives.

### Statistical Analysis

The distribution of sociodemographic characteristics among decedents at the time of death was described using means and proportions according to health care utilization gradients. Overall and per-person health care utilization metrics were calculated for the 2 years, 1 year, 180 days, and 30 days before death using medians and proportions and are presented according to health care utilization gradients for the last year of life. We estimated temporal trends of total health care expenditures among adult deaths from 2005 to 2015 by health care utilization gradient.

Factors associated with being in the top 5% of health care users in the last year of life were assessed by a modified Poisson model.^25^ We chose to model risk directly using a modified Poisson regression because it provides a good approximation of the binomial distribution when the sample is large, and it is less likely than logistic regression to overestimate the relative risk.^26^ We used belonging to the top 5% as the outcome and sex, age, area-level income quintile, and ADG score as the covariates. Associations were calculated with rate ratios (RRs) with corresponding confidence intervals. Statistical significance was set at *P* < .05, and all tests were 2-tailed. All analyses were conducted using SAS Enterprise Guide statistical software, version 7.15 (SAS Institute).

## Results

### Sociodemographic Characteristics by Health Care Utilization Gradient

Sociodemographic characteristics of 966 436 adult decedents (438 038 [50.0%] men; 231 634 [24.0%] living in lowest neighborhood income quintile), stratified by health care utilization gradients (top 5%, top 6%-50%, and bottom 50%) are shown in Table 1. Those in the top 5% were younger, with a mean (SD) age of 71.1 (14.6) years compared with 76.4 (14.96) years for the total cohort. A larger percentage of those in the top 5% were male (26 818 [55.5%] vs 200 965 [46.2%] in the top 6%-50% and 255 255 [52.8%] in the bottom 50%) and had a higher mean (SD) number of ADGs compared with the overall cohort (14.9 [3.6] vs 11.2 [4.4]). In contrast, the distribution of area-level income and education were similar across health care utilization gradients. The number of deaths captured in the cohort per year was similar across years, from 83 227 deaths in 2005 to 95 044 deaths in 2015 (eTable 1 in the Supplement). The major causes of death in the cohort were cancer (287 308 [29.7%]) and diseases of the circulatory system (279 881 [29.0%]) (eTable 2 in the Supplement).

**Table 1.** Sociodemographic Characteristics of All Adult Deaths From 2005 to 2015, Overall and According to Resource Utilization Gradients for the Last Year of Life

Characteristic	No. (%)			
	Overall (N = 966 436)	Resource utilization gradient		
		Top 5% (n = 48 324)	Top 6%-50% (n = 434 899)	Bottom 50% (n = 483 213)
Sex				
Women	483 398 (50.0)	21 506 (44.5)	233 934 (53.8)	227 958 (47.2)
Men	483 038 (50.0)	26 818 (55.5)	200 965 (46.2)	255 255 (52.8)
Age, y				
Mean (SD)	76.4 (14.9)	71.1 (14.6)	78.3 (13.3)	75.2 (15.9)
18-24	5542 (0.6)	349 (0.7)	713 (0.2)	4480 (0.9)
25-34	9444 (1.0)	666 (1.4)	1801 (0.4)	6977 (1.4)
35-44	19 520 (2.0)	1517 (3.1)	5396 (1.2)	12 607 (2.6)
45-54	53 959 (5.6)	3890 (8.0)	18 547 (4.3)	31 522 (6.5)
55-64	103 497 (10.7)	7601 (15.7)	40 758 (9.4)	55 138 (11.4)
65-74	162 318 (16.8)	11 436 (23.7)	71 880 (16.5)	79 002 (16.3)
75-85	314 988 (32.6)	15 766 (32.6)	147 794 (34.0)	151 428 (31.3)
>85	297 168 (30.7)	7099 (14.7)	148 010 (34.0)	142 059 (29.4)
Neighborhood income quintile				
1, Lowest	231 634 (24.0)	11 971 (24.8)	102 617 (23.6)	117 046 (24.2)
2	203 409 (21.0)	10 600 (21.9)	89 643 (20.6)	103 166 (21.4)
3	185 454 (19.2)	8815 (18.2)	84 013 (19.3)	92 626 (19.2)
4	175 248 (18.1)	8423 (17.4)	80 637 (18.5)	86 188 (17.8)
5, Highest	165 864 (17.2)	8154 (16.9)	75 755 (17.4)	81 955 (17.0)
Neighborhood education quintile				
1, Lowest	221 834 (23.0)	10 418 (21.6)	97 944 (22.5)	113 427 (23.5)
2	207 370 (21.5)	9516 (19.7)	91 976 (21.1)	105 878 (21.9)
3	201 661 (20.9)	9989 (20.7)	91 113 (21.0)	100 559 (20.8)
4	180 481 (18.7)	9434 (19.5)	82 848 (19.0)	88 199 (18.3)
5, Highest	144 332 (14.9)	8083 (16.7)	65 972 (15.2)	70 277 (14.5)
Sum of ADGs, mean (SD)	11.2 (4.4)	14.9 (3.6)	12.3 (4.2)	9.8 (4.1)
Weighted ADGs, mean (SD)	27.3 (12.4)	33.4 (11.8)	30.7 (11.4)	23.7 (12.3)
Collapsed ADGs				
Acute minor	878 794 (90.9)	47 476 (98.2)	408 687 (94.0)	422 631 (87.5)
Acute major	904 748 (93.6)	47 777 (98.9)	415 411 (95.5)	441 560 (91.4)
Likely to recur	735 492 (76.1)	44 277 (91.6)	354 228 (81.5)	336 987 (69.7)
Asthma	75 640 (7.8)	5229 (10.8)	36 209 (8.3)	34 202 (7.1)
Chronic medical, unstable	866 822 (89.7)	47 515 (98.3)	405 701 (93.3)	413,606 (85.6)
Chronic medical, stable	768,696 (79.5)	43,770 (90.6)	358,091 (82.3)	366 835 (75.9)
Chronic specialty, stable	78 473 (8.1)	6239 (12.9)	38 409 (8.8)	33 825 (7.0)
Eye or dental	180 015 (18.6)	10 423 (21.6)	80 518 (18.5)	89 074 (18.4)
Chronic specialty, unstable	164 885 (17.1)	10 696 (22.1)	75 411 (17.3)	78 778 (16.3)
Psychosocial	604 159 (62.5)	35 733 (73.9)	309 492 (71.2)	258 934 (53.6)
Prevention, administration	676 627 (70.0)	42 311 (87.6)	333 201 (76.6)	301 115 (62.3)
Pregnancy	4753 (0.5)	450 (0.9)	2322 (0.5)	1981 (0.4)

Abbreviation: ADG, Aggregated Diagnosis Group.

### Health Care Utilization in the Last 2 Years, 1 Year, 180 Days, and 30 Days of Life

Health care utilization prior to death for the overall cohort is described in Table 2. In the last 2 years of life, most individuals (758 770 [78.5%]) had at least 1 acute hospitalization episode of care, with a median (interquartile range [IQR]) length of stay of 8 (5-15) days. Approximately one-third (266 987 [27.6%]) were admitted to the ICU with a median (IQR) length of stay of 69 (33-130) hours in acute care, and almost all (856 026 [88.6%]) had an emergency department visit. The median (IQR) number of visits to primary care and specialist physicians were similar, with 31 (17-53) visits and 34 (13-69) visits, respectively.

**Table 2.** Health Care Utilization Metrics for All Adult Deaths From 2005 to 2015 for the Last 2 Years, 1 Year, 180 Days, and 30 Days of Life, According to Resource Utilization Gradient

Health Care Utilization	Median (IQR)			
	Overall (N = 966 436)	Resource utilization gradient		
		Top 5% (n = 48 324)	Top 6%-50% (n = 434 899)	Bottom 50% (n = 483 213)
**Last 2 y of life**				
Hospitalization episodes of care				
Total hospitalizations, No.	1 947 905	203 559	1 153 423	590 923
≥1 Hospitalizations, No. (%)	758 770 (78.5)	45 736 (94.6)	382 645 (88.0)	330 389 (68.4)
Episodes of care per person, No.	1 (1-3)	3 (2-6)	2 (1-4)	1 (0-2)
Length of stay, d	8 (5-15)	20 (11-36)	10 (6-17)	6 (3-10)
ICU visits				
Total visits, No.	426 869	79 021	219 649	128 199
≥1 ICU admission, No. (%)	266 987 (27.6)	31 099 (64.4)	134 929 (31.0)	100 959 (20.9)
Visits per person, No.	0 (0-1)	1 (0-2)	0 (0-1)	0 (0-0)
Length of stay, h	69 (33-130)	143 (70-317)	76 (41-140)	47 (22-89)
ED visits				
Total visits, No.	3 580 294	283 479	1 958 555	1 338 260
≥1 ED visit, No. (%)	856 026 (88.6)	45 535 (94.2)	401 022 (92.2)	409 469 (84.7)
Visits per person, No.	3 (1-5)	4 (2-8)	3 (2-6)	2 (1-4)
Primary care visits				
Total visits, No.	38 593 836	3 586 798	21 686 232	13 320 806
≥1 Primary care visit, No. (%)	956 176 (98.9)	48 158 (99.7)	434 408 (99.9)	473 610 (98.0)
Visits per person, No.	31 (17-53)	57 (28-106)	41 (27-66)	22 (11-37)
Specialist visits				
Total visits, No.	49 748 655	8 764 734	27 549 679	13 434 242
≥1 Specialist visit, No. (%)	928 231 (96.0)	48 084 (99.5)	425 419 (97.8)	454 728 (94.1)
Visits per person, No.	34 (13-69)	163 (96-238)	54 (22-91)	21 (8-40)
**Last 1 y of life**				
Hospitalization episodes of care				
Total hospitalizations, No.	1 491 243	153 049	895 833	442 361
≥1 Hospitalization, No. (%)	710 035 (73.5)	43 669 (90.4)	364 528 (83.8)	301 838 (62.5)
Episodes of care per person, No.	1 (0-2)	3 (1-4)	2 (1-3)	1 (0-1)
Length of stay, d	8 (4-15)	20 (11-38)	10 (6-17)	6 (3-10)
ICU visits				
Total visits, No.	348 628	66 243	180 352	102 033
≥1 ICU admission, No. (%)	232 948 (24.1)	28 452 (58.9)	118 192 (27.2)	86 304 (17.9)
Visits per person, No.	0 (0-0)	1 (0-2)	0 (0-1)	0 (0-0)
Length of stay, h	69 (31-135)	149 (71-336)	78 (41-147)	45 (19-87)
ED visits				
Total visits, No.	2 536 119	192 192	1 403 282	940 645
≥1 ED visit, No. (%)	805 598 (83.4)	43 007 (89.0)	381 732 (87.8)	380 859 (78.8)
Visits per person, No.	2 (1-3)	3 (1-5)	2 (1-4)	1 (1-3)
Primary care visits				
Total visits, No.	24 836 417	2 518 507	14 336 923	7 980 987
≥1 Primary care visit, No. (%)	948 717 (98.2)	47 877 (99.9)	433 714 (99.7)	467 126 (96.7)
Visits per person, No.	18 (10-35)	40 (16-80)	26 (15-46)	13 (6-23)
Specialist visits				
Total visits, No.	35 113 183	6 732 801	19 842 381	8 538 001
≥1 Specialist visit, No. (%)	898 453 (93.0)	47 781 (98.9)	415 524 (95.5)	435 148 (90.1)
Visits per person, No.	22 (7-49)	124 (68-188)	39 (13-68)	13 (4-27)
**Last 180 d of life**				
Hospitalization episodes of care				
Total hospitalizations, No.	1 153 854	94 694	670 755	388 405
≥1 Hospitalization, No. (%)	662 628 (68.6)	38 793 (80.3)	338 038 (77.7)	285 797 (59.1)
Episodes of care per person, No.	1 (0-2)	2 (1-3)	1 (1-2)	1 (0-1)
Length of stay, d	8 (4-15)	19 (9-37)	10 (5-18)	6 (3-10)
ICU visits				
Total visits, No.	290 261	47 425	147 530	95 306
≥1 ICU admission, No. (%)	205 365 (21.2)	22 908 (47.4)	100 757 (23.2)	81 700 (16.9)
Visits per person, No.	0 (0-0)	0 (0-1)	0 (0-0)	0 (0-0)
Length of stay, h	69 (30-139)	162 (71-367)	83 (41-159)	44 (18-86)
ED visits				
Total visits, No.	1 749 134	106 406	935 668	707 060
≥1 ED visit, No. (%)	750 558 (77.7)	37 276 (77.1)	355 188 (81.7)	358 094 (74.1)
Visits per person, No.	1 (1-2)	2 (1-3)	2 (1-3)	1 (0-2)
Primary care visits				
Total visits, No.	16 561 145	1 543 472	9 669 778	5 347 895
≥1 Primary care visit, No. (%)	935 451 (96.8)	47 015 (97.3)	431 748 (99.3)	456 688 (94.5)
Visits per person, No.	11 (6-23)	24 (8-48)	16 (8-31)	8 (4-15)
Specialist visits				
Total visits, No.	24 911 856	4 551 779	14 049 305	6 310 772
≥1 Specialist visit, No. (%)	859 752 (89.0)	46 900 (97.1)	400 257 (92.0)	412 595 (85.4)
Visits per person, No.	14 (4-35)	75 (29-135)	24 (7-49)	9 (2-20)
**Last 30 d of life**				
Hospitalization episodes of care				
Total hospitalizations, No.	558 561	23 254	265 940	269 367
≥1 Hospitalization, No. (%)	475 574 (49.2)	18 826 (39.0)	220 309 (50.7)	236 439 (48.9)
Episodes of care per person, No.	0 (0-1)	0 (0-1)	1 (0-1)	0 (0-1)
Length of stay, d	6 (3-11)	8 (4-15)	7 (4-13)	5 (2-9)
ICU visits				
Total visits, No.	174 975	11 185	80 836	82 954
≥1 ICU admission, No. (%)	143 225 (14.8)	8376 (17.3)	62 032 (14.3)	72 817 (15.1)
Visits per person, No.	0 (0-0)	0 (0-0)	0 (0-0)	0 (0-0)
Length of stay, h	59 (23-124)	101 (39-223)	87 (37-174)	41 (16-84)
ED visits				
Total visits, No.	696 385	23 312	298 370	374 703
≥1 ED visit, No. (%)	528 658 (54.7)	16 916 (35.0)	223 262 (51.3)	288 480 (59.7)
Visits per person, No.	1 (0-1)	0 (0-1)	1 (0-1)	1 (0-1)
Primary care visits				
Total visits, No.	6 103 335	375 130	3 393 046	2 335 159
≥1 primary care visit, No. (%)	856 679 (88.6)	39 659 (82.1)	407 616 (93.7)	409 404 (84.7)
Visits per person, No.	4 (1-9)	5 (1-11)	5 (2-11)	3 (1-6)
Specialist visits				
Total visits, No.	10 062 164	1 327 245	5 423 675	3 311 244
≥1 specialist visit, No. (%)	699 042 (72.3)	41 599 (86.1)	326 249 (75.0)	331 194 (68.5)
Visits per person, No.	4 (0-14)	15 (3-42)	5 (1-17)	3 (0-10)

Abbreviations: ED, emergency department; ICU, intensive care unit; IQR, interquartile range.

In the last 30 days of life, 143 225 decedents (14.8%) were admitted to the ICU, spending a median (IQR) of 59 (23-124) hours in acute care. In addition, most visited a primary care physician (856 679 [88.6%]; median [IQR] visits, 4 [1-9]) and a specialist (699 042 [72.3%]; median [IQR] visits, 4 [0-14]). In terms of proximity to death, 475 574 decedents (49.2%) had at least 1 hospitalization episode of care in the last 30 days of life, 662 628 (68.6%) in the last 180 days, 710 035 (73.5%) in the last year, and 758 770 (78.5%) in the last 2 years. Similarly, the proportion that visited the emergency department was 528 658 (54.7%) in the last 30 days of life, 750 558 (77.7%) in the last 180 days, 805 598 (83.4%) in the last year, and 856 026 (88.6%) in the last 2 years. The nominal difference in percentage demonstrates that a substantial portion of health care use occurred toward the end of life.

### Health Care Utilization Metrics by Resource Utilization Gradients

Table 2 presents health care utilization metrics at the end of life according to resource utilization gradients. In the last 2 years of life, among those who experienced a hospitalization, individuals in the top 5% had a median (IQR) of 3 (2-6) episodes of care per person, compared with 1 (0-2) episode of care among individuals in the bottom 50%. In the same period, approximately two-thirds of those in the top 5% experienced an ICU admission (31 099 [64.4%]) with a median (IQR) length of stay of 143 (70-317) hours; in comparison, approximately one-fifth of individuals in the bottom 50% (100 959 [20.9%]) had an ICU admission, with a median (IQR) length of stay of 47 (22-89) hours. The proportion of individuals visiting the emergency department was slightly higher among the top 5% compared with other utilization groups in the last 2 years (top 5%, 45 535 [94.2%]; top 6%-50%, 401 022 [92.2%]; bottom 50%, 409 469 [84.7%]) and 1 year (top 5%, 43 007 [89.0%]; top 6%-50%, 381 732 [87.8%]; bottom 50%, 380 859 [78.8%]) of life. In contrast, in the last 30 days of life, more than half of individuals in the top 6% to top 50% (223 262 [51.3%]) and bottom 50% (288 480 [59.7%]) visited an emergency department, compared with approximately one-third of individuals in the top 5% (16 916 [35.0%]). In the last 2 years of life, the median (IQR) number of primary care visits was 57 (28-106) among the top 5% compared with 22 (11-37) among the bottom 50%. The median (IQR) number of specialist visits over this period was 163 (96-238) among the top 5% compared with 21 (8-40) among the bottom 50%.

### Factors Associated With High Resource Utilization Prior to Death

In the Poisson model (Table 3), significant risk reductions for high resource utilization (ie, top 5%) in the last year of life were observed among women compared with men (RR, 0.90; 95% CI, 0.88-0.91) and among older age groups; the RR was 0.21 times lower in decedents older than 85 years compared with those aged 18 to 24 years (RR, 0.21; 95% CI, 0.19-0.23) after adjusting for income and ADGs (Table 3). No meaningful associations were observed between individuals in the highest area income quintile compared with individuals in the lowest quintile (RR, 1.02; 95% CI, 0.99-1.05) after adjusting for sex, age, and ADGs. The associations between high income (ie, quintile 5) and low income (ie, quintile 1) remained null in the sex-segregated models, in which the confidence interval included the null value.

**Table 3.** Rate Ratios for Being in the Top 5% of Health Care Users in the Last Year of Life, Among All Adult Decedents in Ontario, 2005 to 2013^a^

Variable	All		Men		Women	
	RR (95% CI)	*P* value	RR (95% CI)	*P* value	RR (95% CI)	*P* value
Sex^b^						
Men	1 [Reference]	NA	NA	NA	NA	NA
Women	0.90 (0.88-0.91)	<.001	NA	NA	NA	NA
Age^b^						
18-24	1 [Reference]	NA	1 [Reference]	NA	1 [Reference]	NA
25-34	0.78 (0.68-0.89)	<.001	0.81 (0.68-0.97)	.02	0.74 (0.61-0.90)	.002
35-44	0.73 (0.65-0.82)	<.001	0.68 (0.57-0.79)	<.001	0.78 (0.66-0.93)	.004
45-54	0.63 (0.57-0.71)	<.001	0.62 (0.53-0.72)	<.001	0.65 (0.55-0.76)	<.001
55-64	0.60 (0.54-0.67)	<.001	0.61 (0.52-0.70)	<.001	0.60 (0.51-0.70)	<.001
65-74	0.52 (0.46-0.57)	<.001	0.53 (0.46-0.61)	<.001	0.50 (0.43-0.59)	<.001
75-85	0.36 (0.32-0.40)	<.001	0.38 (0.33-0.44)	<.001	0.33 (0.29-0.39)	<.001
>85	0.21 (0.19-0.23)	<.001	0.26 (0.22-0.30)	<.001	0.17 (0.15-0.20)	<.001
Area income quintile^b^						
1, lowest	1 [Reference]	NA	1 [Reference]	NA	1 [Reference]	NA
2	1.04 (1.01-1.06)	<.001	1.02 (0.99-1.06)	.22	1.05 (1.01-1.09)	.01
3	0.97 (0.94-1.00)	.03	0.97 (0.93-1.00)	.08	0.97 (0.93-1.01)	.16
4	0.98 (0.96-1.01)	.20	0.99 (0.95-1.03)	.52	0.97 (0.93-1.01)	.16
5, highest	1.02 (0.99-1.05)	.18	1.04 (1.00-1.08)	.03	0.98 (0.94-1.03)	.42
Missing	1.46 (1.32-1.62)	<.001	1.67 (1.47-1.90)	<.001	1.17 (0.96-1.40)	.10
ADG score, per 1-unit increase^b^	1.27 (1.27-1.28)	<.001	1.28 (1.27-1.28)	<.001	1.27 (1.26-1.27)	<.001

Abbreviations: ADG, Aggregated Diagnosis Group; NA, not applicable; RR, rate ratio.

^a^ Rate ratios were calculated using modified Poisson model.

^b^ Likelihood ratio *P* values for the overall variables in type 3 analysis where *P* < .001.

### Hospital Deaths by Resource Utilization Gradients

Table 4 displays trends in the percentage of deaths that occurred in hospital by resource utilization gradients. Overall, deaths in hospital decreased from 37 984 (45.6%) in 2005 to 39 474 (41.5%) in 2015. Throughout the study period, a total of 29 292 of 48 324 deaths (60.4%) and 203 792 of 483 213 (42.2%) occurred in the hospital among those in the top 5% and the bottom 50%, respectively, without much variation during the study period. Among the top 6% to top 50% resource gradient, deaths in hospital decreased from 14 975 of 28 792 (52.0%) in 2005 to 18 569 of 46 859 (39.6%) in 2015.

**Table 4.** Percentage of Deaths in Hospital Among All Adult Deaths From 2005 and 2015 and According to Resource Utilization Gradients

Resource utilization gradient^a^	Deaths in hospital, No./total No. (%) by year										
	2005	2006	2007	2008	2009	2010	2011	2012	2013	2014	2015
Overall	37 984/83 227 (45.6)	38 200/82 445 (46.3)	39 424/85 034 (46.4)	40 042/85 822 (46.7)	40 099/86 222 (46.5)	39 964/87 216 (45.8)	39 407/87 764 (44.9)	38 613/88 510 (43.6)	38 978/91 304 (42.7)	39 996/93 848 (42.6)	39 474/95 044 (41.5)
Top 5%	1367/2411 (56.7)	1695/2867 (59.1)	2383/4026 (59.2)	2948/4789 (61.6)	3160/5043 (62.7)	3095/4963 (62.4)	2914/4736 (61.5)	2706/4523 (59.8)	2998/5027 (59.6)	3027/5003 (60.5)	2999/4936 (60.8)
Top 6%-50%	14 975/28 792 (52.0)	16 489/31 601 (52.2)	18 781/36 311 (51.7)	18 847/36 513 (51.6)	18 551/37 367 (49.6)	18 640/40 394 (46.1)	18 266/41 810 (43.7)	18 403/43 698 (42.1)	18 485/44 954 (41.1)	19 091/46 600 (41.0)	18 569/46 859 (39.6)
Bottom 50%	21 642/52 024 (41.6)	20 016/47 977 (41.7)	18 260/44 697 (40.9)	18 247/44 520 (41.0)	18 388/43 812 (42.0)	18 229/41 859 (43.5)	18 227/41 218 (44.2)	17 504/40 289 (43.4)	17 495/41 322 (42.3)	17 878/42 245 (42.3)	17 906/43 249 (41.4)

^a^ Based on the year before death.

### Temporal Trends in Health Care Expenditures According to Resource Utilization Gradients

Total health care expenditures in the last 2 years of life increased in Ontario from CAD$5.12 billion (US $3.83 billion) in 2005 to CAD$7.84 billion (US $5.86 billion) in 2015, an increase of approximately 35%. Similarly, expenditures during this period increased from CAD$3.59 billion (US $2.69 billion) to CAD$5.34 billion (US $4.01 billion) in the last year of life, an increase of 33%. In the last 180 days of life, expenditures increased from CAD$2.53 billion (US $1.90 billion) to CAD$3.67 billion (US $2.75 billion), a 31% increase, and for the last 30 days of life, they increased from CAD$1.04 billion (US $0.78 billion) to CAD$1.43 billion (US $1.07 million), a 27% increase (Figure, A). Mean per-person spending in the last 2 years of life increased among the top 5% from CAD$273 820 (95% CI, CAD$269 935 to CAD$277 760) (US $205 365; 95% CI, US $202 451 to US $208 320) in 2005 to CAD$295 183 (95% CI, CAD$291 811 to CAD$298 593) (US $221 387; 95% CI, US $218 858 to US $223 945) in 2015. In the same period, mean per-person spending in the bottom 50% decreased from CAD$33 489 (95% CI, CAD$33 210 to CAD$33 771) (US $25 117; 95% CI, US $24 908 to US $25 328) in 2005 to CAD$31 148 (95% CI, CAD$30 871 to CAD$31 427) (US $23 361; 95% CI, US $23 153 to $23 570) in 2015 (Figure, B). In the last 2 years of life, mean (SD) per-person spending for acute hospital care increased from CAD$4839 (CAD$ 14 053) (US $3629 [US $10 540]) in 2005 to CAD$6572 (CAD$19 722) (US $4928 [US $14 792]) in 2015 (Figure, C).

**Figure.**
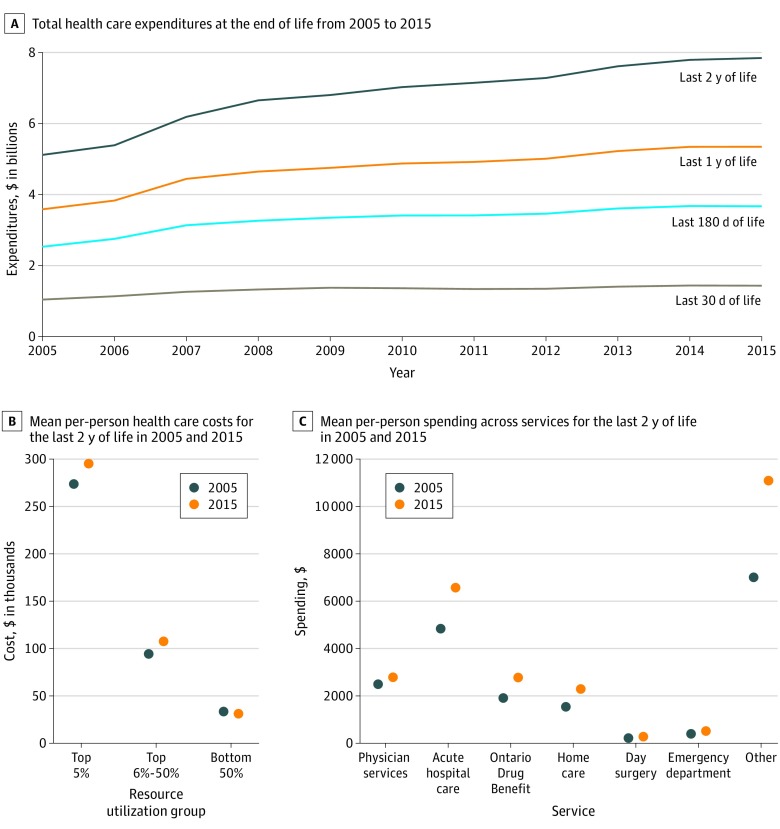
Distribution of Health Care Spending Before Death Expenditures were calculated in Canadian dollars for the year 2015.

## Discussion

This study examined population-wide health care utilization and costs at the end of life in the universal health care system of Ontario, which accounts for 40% of Canada. Our unique focus on health care utilization gradients and trends of health care use at the end of life was enabled by a mortality database that contained all deaths registered in Ontario during 11 years, linked with health administrative data. We demonstrated that overall health care expenditures in Ontario for the last 2 years of life increased by 35% from 2005 to 2015, with the largest proportional increase in average per-person spending observed in the top 5% and top 6% to top 50% of health care users. We demonstrated higher end-of-life utilization of health care services among those in the top 5% compared with the overall cohort for hospitalization episodes of care, ICU visits, emergency department visits, and physician visits. Exceptions to this pattern were identified for the last 30 days of life, in which utilization of certain services, such as emergency department visits, were higher among the top 6% to top 50% and bottom 50% of health care users than among the top 5%. However, the observed reduced utilization of these services could have been the result of individuals in the highest cost group already being admitted to a hospital in their last 30 days of life. Several studies have reported population-wide health care utilization prior to death,^19,27,28^ but they have not looked at differences among health care use gradients.

The study showed that in the last year of life, 74% of residents of Ontario had a hospitalization episode of care and 24% spent time in the ICU. Comparable patterns of end-of-life health care utilization have been reported in other high-income countries. For example, an Australian study looking at hospital-based services used by adults during the last year of life reported slightly higher rates of hospitalization (84%) and lower rates of ICU visits (12%).^27^ In the United States, ICU visit rates in the last month of life ranged from 24% to 29% among Medicare beneficiaries aged 66 years and older compared with 21% in our cohort.^29^

We observed a negatively linear association between older age groups and being in the top 5% of health care users in the last year of life, especially among men. A similar pattern of lower expenditures among older age groups in the last year of life was reported in the US Medicare population of adults aged 65 and older.^30^ In our analysis, we did not see meaningful associations for area-level income quintiles and high health care utilization in the last year of life after adjusting for sex, age, and ADGs. Similar findings were observed in a retrospective cohort analysis of health care use among deaths in Ontario from 2010 to 2013, in which total costs did not vary by neighborhood income quintile.^19^ In contrast, among the US Medicare population, individuals in the lowest-income area had slightly higher expenditures in the last year of life compared with those living in the highest-income areas.^30^ Furthermore, in a study of health care spending in the last year of life in the province of British Columbia, Canada, the highest 2 household income quintiles were shown to have approximately 4% less health care spending than those in the lowest income quintile.^31^ The differential income associations observed in these studies could be attributed to health system differences in access to health care services in the jurisdictions under study and differences in ecological-level vs individual-level income measures used.

A larger percentage of deaths occurred in hospital in our cohort compared with Switzerland, where it was reported that 34% of deaths were in a hospital,^28^ and in the United States, where deaths in acute care hospitals ranged from 25% to 33% among decedents older than 66 years.^29^ Furthermore, we observed that the proportion of deaths in hospital among the top 5% and bottom 50% of health care users in the last year of life was stable over the study period. The observed high-intensity care near the end of life and high percentage of deaths in hospitals highlights a need for a societal-level discussion about approaches to end-of-life care in Ontario.

We observed that high health care utilization was associated with multimorbidity, as measured by ADGs, and that hospital-centered care was the typical trajectory at the end of life. This points to the need to design appropriate integrated care strategies that could support patients at the end of life to be discharged from the hospital and receive care and management for their conditions through home care or long-term care services.

### Limitations

It is important to note some limitations to our study. First, our study used ecological-level indicators of socioeconomic status based on postal code information at the time of death, which may have provided lower estimates of income gradients in health care utilization.^32^ Second, our database only included services covered by the provincial health care payee and not services that may be covered by supplemental insurance or paid for out of pocket (ie, nursing, personal care, medications, and therapy). Third, comprehensive recommendations regarding end-of-life care are difficult to make in the absence of information on the appropriateness of care and use of potentially avoidable health services, which were out of scope for this study. Nonetheless, the findings support understanding of end-of-life health care trends in a universal health care system.

## Conclusions

This study reported on health care utilization in the 2 years before death with a focus on the characterization of high-cost users. It identified patterns of high utilization of health care services before death and a large proportion of deaths in hospital, with variation across health care utilization gradients. The findings suggest a trajectory of hospital-centered care prior to death in Ontario.

## Notes

Supplement. **eTable 1. **Proportion of Deaths in the Cohort per Year **eTable 2. **Causes of Death from 2005 to 2015
