# Supplementary material for: Population-Based Estimates of Health Care Utilization and Expenditures by Adults During the Last 2 Years of Life in Canada’s Single-Payer Health System
Source: JAMA Netw Open. 2020 Apr 1;3(4):e201917. doi: 10.1001/jamanetworkopen.2020.1917 (PMC7113729; doi:10.1001/jamanetworkopen.2020.1917)
Supplement: Supplement. — eTable 1. Proportion of Deaths in the Cohort per Year eTable 2. Causes of Death from 2005 to 2015 [file jamanetwopen-3-e201917-s001.pdf]

Supplementary Online Content

Rosella LC, Kornas K, Bornbaum C, et al. Population-based estimates of health care utilization and expenditures by adults during the last 2 years of life in Canada's single-payer health system. *JAMA Netw Open*. 2020;3(4):e201917. doi:10.1001/jamanetworkopen.2020.1917

eTable 1. Proportion of Deaths in the Cohort per Year

eTable 2. Causes of Death from 2005 to 2015

This supplementary material has been provided by the authors to give readers additional information about their work.

© 2020 Rosella LC et al. *JAMA Network Open.*

eTable 1. Proportion of Deaths in the Cohort per Year

	Number of Deaths	
	**n**	**%**
**2005**	83,227	8.6
**2006**	82,445	8.5
**2007**	85,034	8.8
**2008**	85,822	8.9
**2009**	86,222	8.9
**2010**	87,216	9.0
**2011**	87,764	9.1
**2012**	88,510	9.2
**2013**	91,304	9.4
**2014**	93,848	9.7
**2015**	95,044	9.8
**Overall**	966,436	100

© 2020 Rosella LC et al. *JAMA Network Open.*

eTable 2. Causes of Death from 2005 to 2015 (n=966,436)

Cause of Death	Number of Deaths	
	n	%
**Cancer**	287,308	29.7
**Diseases of the Circulatory System**	279,881	29.0
**Diseases of the Respiratory System**	87,893	9.1
**External Causes of Morbidity and Mortality**	57,508	6.0
**Diseases of the Nervous System**	43,294	4.5
**Diseases of the Digestive System**	40,085	4.1
**Endocrine, Nutritional and Metabolic Diseases**	38,701	4.0
**Other**	131,766	13.6
**Missing**	598	<0.0

© 2020 Rosella LC et al. *JAMA Network Open.*
